# Phytochemical profiling and network pharmacology-based prediction of *Pelargonium graveolens* essential oil in the context of Guillain-Barré syndrome

**DOI:** 10.1038/s41598-025-13101-4

**Published:** 2025-08-05

**Authors:** Abdullah Haikal, Abdelrahman Hamdi, Aya M. Almatary

**Affiliations:** 1 Department of Pharmacognosy, Faculty of Pharmacy, Mansoura University, Mansoura, 35516 Egypt; 2 Department of Pharmaceutical Organic Chemistry, Faculty of Pharmacy, Mansoura University, Mansoura, 35516 Egypt; 3 Department of Pharmaceutical Chemistry, Faculty of Pharmacy, Horus University-Egypt, New Damietta, 34518 Egypt

**Keywords:** *Pelargonium graveolens*, Essential oil, Guillain-Barré syndrome, TNF-α, Network pharmacology, And molecular modelling, Chemical biology, Computational biology and bioinformatics, Plant sciences

## Abstract

Guillain-Barré syndrome (GBS) is a rare autoimmune disorder with limited treatment options. This study investigates the chemical composition of *Pelargonium graveolens* essential oil and explores its potential interaction with GBS-related molecular targets using *in-silico* tools. Essential oil was analyzed using GC–MS. Identified components were subjected to network pharmacology analysis and molecular docking to assess binding affinities with GBS-relevant proteins, particularly TNF-α. Thirty compounds were identified, with citronellol (26.74%) and geraniol (20.28%) as major components. Comparative phytochemical analysis showed consistency with global *P. graveolens* chemotypes. Docking results suggest high binding affinity of citronellyl and geranyl derivatives toward TNF-α. These findings support further investigation of *P. graveolens* oil as a potential modulator of inflammatory pathways in GBS.

## Introduction

Guillain-Barré Syndrome (GBS), also known as Acute Inflammatory Demyelinating Polyneuropathy, is an autoimmune disorder that targets the peripheral nervous system. It is primarily triggered by an aberrant immune response to a preceding infectious agent^1^. Clinically, flaccid paralysis, gradual, symmetrical muscular weakening, and varied degrees of sensory impairment are the hallmarks of GBS^2^. It is known that immune-mediated inflammation is a major factor in the development and progression of GBS, even if the exact pathophysiological processes of the condition are still unclear^3,4^. According to available data, inflammatory cytokines play a major role in both localized and systemic immune responses that cause peripheral nerve demyelination and axonal damage^5^. Based on experimental research, these cytokines encourage effector immune cells to be drawn to peripheral nerve roots, which results in the generation of reactive oxygen species, nitric oxide, and other mediators of nerve damage^6^. Patients with GBS have been shown to have elevated amounts of interleukin-6 (IL-6) and tumor necrosis factor-alpha (TNF-α) in their blood, which are correlated with the severity of the illness and its clinical symptoms^3^. Furthermore, elevated TNF-α expression has been suggested as a possible biomarker and mechanistic factor in the pathophysiology of GBS^7^. The majority of modern medical care uses supportive and symptomatic therapy^8,9^. In addition to immunoglobulin therapy, plasmapheresis therapy, hormone therapy, and cerebrospinal fluid filtration, intravenous immunoglobulin and plasma exchange are equally effective treatments for Guillain-Barré Syndrome^10–13^. There is currently no specific medication to treat Guillain-Barré Syndrome, and classical therapies often involve etiological programs in addition to neurotrophic and anti-inflammatory strategies. Furthermore, high-dose immunoglobulin administered intravenously has been shown to provide positive results; nevertheless, it is currently costly and may cause adverse consequences. Effective supplemental therapy techniques, such as those used in traditional Chinese medicine, are still needed to treat this illness. There have been reports of special benefits of medicinal plants in treating neurodegenerative diseases, demyelinating disorders, and autoimmune neuritis^14,15^.

There were about 750–800 species in the Geraniaceae family, which included three major genera: Erodium, Geranium, and Pelargonium. With over 300 species, the genus *Pelargonium* is extensively dispersed in South Africa. It has recently been brought to North Africa, Asia, and Europe^16,17^. Numerous *Pelargonium* species are used as traditional medicines to cure a variety of illnesses, many of which are bacterial or fungal diseases. Pelargonium species are used in herbal treatments to cure wounds, abscesses, fever, colic, nephritis, colds, and sore throats. They are also used as anthelmintic and insecticidal agents, to reduce urination, and to stimulate milk production. It was well known that *Pelargonium* species might help with diarrhea and dysentery^18–20^.

The essential oil of *Pelargonium graveolens* (Thunb.) L'Hér, an aromatic herb, is used as a fragrant ingredient in food and beverage production, perfumery, and as an antidepressant and antimicrobial. Together with its diuretic qualities, this essential oil’s astringent and chemostatic effects, circulatory regulation, lymphatic system stimulation, and adrenal gland stimulation make it a great tool for combating cellulite and fluid retention in the body. It is used to treat a variety of skin conditions and maintain oral hygiene because of its antiseptic properties^21^. Citronellol and geraniol are two important bioactive chemicals found in *P. graveolens* essential oil that may be linked to these activities^22^. Structure-based drug design one of the most commonly used techniques to develop new medications, depends on identifying and describing biological targets, mainly proteins, and then matching those targets with small molecule compounds that have a significant affinity for them^23–26^. Therefore, utilizing the new technique of network pharmacology and *in-silico* analysis, we are attempting to phytochemically examine the essential oils of *P. graveolens* and explore their potential to target GBS in the context of TNF-α biomarker.

## Materials and methods

### Plant material

Fresh aerial parts of *Pelargonium graveolens* (Thunb.) L'Hér (Rose-scented geranium) were collected in July from the medicinal plant station, Pharmacognosy Department, Faculty of Pharmacy, Mansoura University, Dakahlia, Egypt. Prof. Ibrahim Mashaly, Professor of ecology and systematic botany at Mansoura University’s Faculty of Science, verified the identity of the plant. A voucher specimen (PGA4.2024) has been placed at Mansoura University’s Faculty of Pharmacy, Pharmacognosy Department Herbarium^27,28^. The plant collected according to IUCN Policy Statement on Research Involving Species at Risk of Extinction and the Convention on International Trade in Endangered Species of Wild Fauna and Flora.

### Hydro-distillation of the essential oil

To obtain oil, 250 g of fresh *Pelargonium graveolens* aerial parts were crushed into small pieces and hydro-distilled for three hours using Clevenger apparatus. Anhydrous sodium sulphate was used to collect and dehydrate the resulting oil, which was then stored at low temperatures in a sealed vial for GC/MS analysis and biological research.

### Gas chromatography-mass spectrometry (GC/MS) analysis

Utilizing a Thermo Scientific Trace GC-ISQ mass spectrometer, which has a TG-5MS capillary column with 30 m long, 0.25 mm in diameter, and 0.25 m in film thickness, along with an A3000 autosampler, the GC/MS analysis was carried out. Gradient mode (10 °C/minute) was used to plan the temperature between 50 °C and 280 °C. The injector, interface, and source all have temperatures of 220 °C, 220 °C, and 200 °C, respectively. Mass spectrometer set at 70 eV in EI mode. Using a 50–600 amu mass scan, one μL of diluted material was injected in splitless mode. Helium was employed as the carrier gas (1 mL/min). In order to calculate each molecule’s retention index and identify the oil components based on their retention indices, a mixture of fatty acid methyl esters (C5–C20) was instantly fed into the GC injector under the previously mentioned temperature program. Van Den Dool’s method was used to compute all component retention indices. The components’ identities were further confirmed by comparing their base peak and mass spectral fragmentation patterns with those reported in the literature or in the mass spectral databases NIST and ChemStation data system^29,30^.

### Network pharmacology study

#### Clustering of geranium oil and Guillain-Barré syndrome target genes

The Geranium oil-related target genes were clustered depending on chemical similarities and pharmacophore models via the swisstargetpredict database.

Guillain-Barré syndrome (GBS)-related target genes were collected from three databases, namely, Disegent, GeneCard (https://www.genecards.org/) and OMIM (Online Mendelian Inheritance in Man, https://omim.org/ search/advanced/geneMap). Potential target genes (i.e., overlapping target genes) of geranium oil therapy for GBS were acquired through the Veeny 2.1 (https://bioinfogp.cnb. csic.es/tools/venny/) intersection.

#### Protein–Protein Interaction (PPI) network map of the Geranium oil and Guillain-Barré syndrome -potential target genes

A PPI network map was constructed for the co-expression, fusion, neighborhood, and co-localization of potential target genes with predicted gene interactions^31^. The name of the potential target genes was entered via the STRING database (https://string-db.org/) with “Homo sapiens” being selected. Each node represents a protein in the PPI network map, and each edge represents a functional association between potential target genes.

### Molecular docking study

Docking the oil active components within the active site of TNF-α were explored using MOE (Molecular Operating Environment) software. The co-crystallized ligand, 6,7-dimethyl-3-[(methyl{2[methyl({1-[3-(trifluoromethyl) phenyl]-1H-indol-3-yl}methyl) amino] ethyl}amino)methyl]-4H-chromen-4-one with its PDB file was downloaded from the protein data bank (PDB ID: 2AZ5). Molecular docking simulation was performed in Computational Chemistry and Molecular Modeling Lab, Pharmaceutical Organic Chemistry Department, Faculty of Pharmacy, Mansoura University using "Molecular Operating Environment (MOE) 2024.06." https://www.chemcomp.com/en/index.htm.

## Result and discussion

### Essential oil composition of *Pelargonium graveolens* (Thunb.) L'Hér aerial parts

**Fig. 1:**
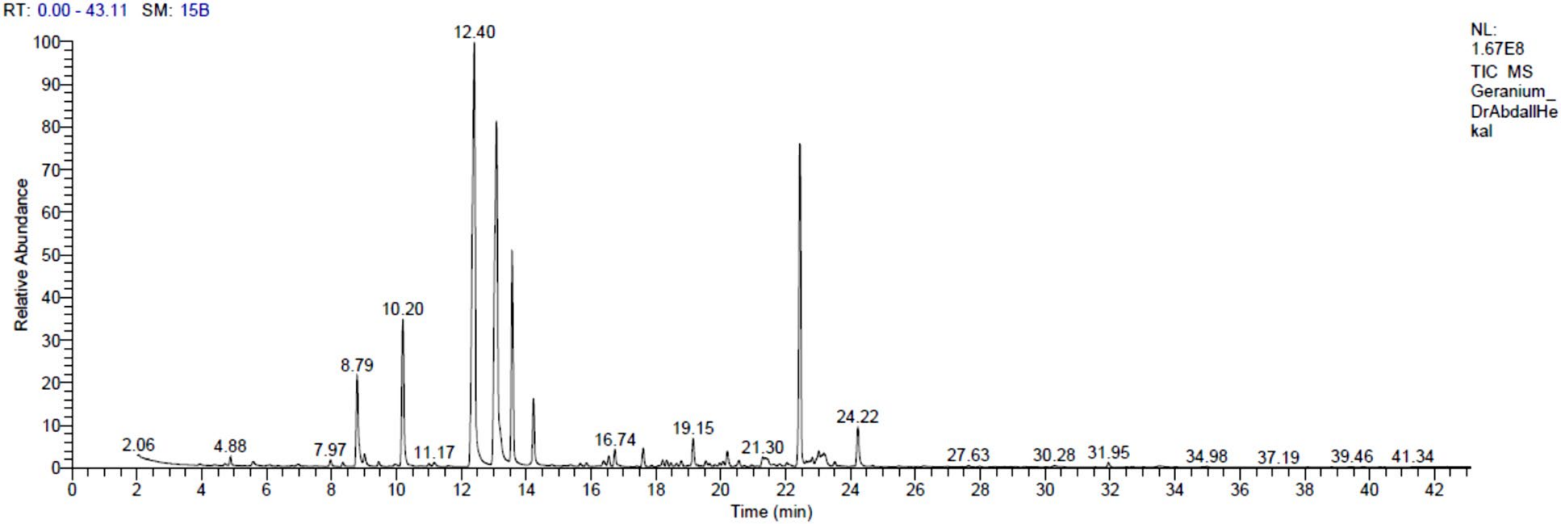
Gas chromatogram of the essential oil from *Pelargonium graveolens* (Thunb.) L' Hér aerial parts.

*Pelargonium graveolens* aerial parts were utilised to extract a yellow essential oil that was lighter than water and had a yield of 0.17% *v/w*. Table 1 lists the components of the oil along with their percentages and retention indices. Thirty compounds (99.44%) have been identified. Monoterpenes, which make up the majority of the oil’s constituents, account for 76.34% of the mixture, while sesquiterpenes make up 23.1%. While 10-epi-γ-Eudesmol (17.14%) is the primary component of sesquiterpenes, the predominant components of monoterpenes are citronellol (26.74%), geraniol (20.28%), citronellyl formate (9.30%), and menthone (7.03%). About 76.0% of the identified monoterpenes are oxygenated monoterpenes, whereas 19.12% are oxygenated sesquiterpenes. Monoterpene ester compounds constitute 16.46% found in *P. graveolens* (Table 1, Fig. 1). The sole aromatic volatile compound identified in the components is calamenene. The oxygenated monoterpenes citronellol (26.74%) and geraniol (20.28%) make up the majority of the essential oil found in *P. graveolens* aerial parts.

**Table 1:** Essential oils’ composition of *Pelargonium graveolens* (Thunb.) L' Hér aerial parts.

Peak No	Retention time	Literature R_*I*_^32^	Calculated R_*I*_	M^+^ peak	Base peak	Peak area %	Identified compounds	Structure
1	4.88	939	937	136.2	93.0	0.34	*α*-Pinene	
2	7.97	1072	1070	170.2	59.0	0.31	cis-Linalool oxide	
3	8.79	1096	1093	154.2	71.0	4.52	Linalool	
4	9.02	1108	1110	154.2	139.0	0.44	cis-Rose oxide	
5	9.45	1125	1127	154.2	139.0	0.22	trans-Rose oxide	
6	10.20	1152	1151	154.2	112.0	**7.03**	**Menthone**	
7	12.41	1225	1225	156.3	41.0	**26.74**	**Citronellol**	
8	13.09	1252	1251	154.2	41.0	**20.28**	**Geraniol**	
9	13.57	1273	1270	184.3	41.0	**9.30**	**Citronellyl formate**	
10	14.22	1298	1300	182.3	69.0	2.88	Geranyl formate	
11	16.39	1376	1372	204.4	161.0	0.26	*α*-Copaene	
12	16.55	1381	1383	196.3	69.0	0.43	Geranyl acetate	
13	16.74	1388	1389	204.4	81.0	0.74	*β*-Bourbonene	
14	17.61	1408	1411	204.4	41.0	0.86	Caryophyllene	
15	18.20	1436	1433	204.4	121.0	0.30	*γ*-Elemene	
16	18.33	1444	1444	204.4	105.0	0.26	Guaia-6,9-diene	
17	18.78	1460	1461	204.4	41.0	0.30	Alloaromadendrene	
18	19.15	1475	1477	210.3	69.0	1.27	Geranyl propionate	
19	19.54	1485	1484	204.4	161.0	0.30	Germacrene D	
20	20.07	1522	1520	202.3	159.0	0.19	Calamenene	
21	20.20	1523	1522	204.4	161.0	0.77	*δ*-Cadinene	
22	21.29	1578	1577	220.4	43.0	0.50	Spathulenol	
23	21.38	1583	1583	220.4	41.0	0.38	Caryophyllene oxide	
24	21.45	1600	1601	222.0	161.0	0.23	Guaiol	
25	22.44	1623	1622	222.0	189.0	**17.14**	**10-epi-*γ*-Eudesmol**	
26	22.82	1646	1644	222.0	43.0	0.29	Cubenol	
27	23.02	1648	1645	220.4	43.0	0.39	Agarospirol	
28	23.17	1657	1656	238.4	55.0	0.68	Citronellyl tiglate	
29	23.51	1678	1679	220.4	41.0	0.19	Aromadendrene oxide II	
30	24.22	1696	1699	236.3	83.0	1.90	Geranyl tiglate	
						99.44		

Bold values point out the major components.

We could observe a number of observations by comparing the percentages of components in the plant oil indicated above with the percentages of compounds in the same oil from other geographic locations. The main components of the oil, in varying percentages, are oxygenated monoterpenes citronellol and geraniol, which are also gathered in Egypt from the Faculty of Pharmacy’s Experimental Station of Medicinal Plants at Cairo University. In addition to the European nations of Bosnia and Herzegovina and Serbia, oil extracted from plants in Africa (such as Morocco and Tunisia) or Asia (such as India and Tajikistan) has a high concentration of the oxygenated monoterpenes citronellol and geraniol, albeit in varying amounts. In contrast, a different research conducted on the campus of Zagazeg University in Egypt found that *P. graveolens* essential oil had a significant concentration of oxygenated monoterpenes, including citronellol (27.67%) and cis-menthone (10.23%). The majority of the constituents in oil from Algeria, Iran, and Palestine are oxygenated monoterpene citronellol and monoterpene ester citronellyl formate. We observed that the oxygenated monoterpenes geraniol (42.3%) and linalool (16.4%) make up the majority of the oil’s constituents when we landed in Brazil, South America. The two main constituents of Iraqi oil are menthol (9.61%) and citronellyl isovalerate ester (10.41%). Lastly, the oil from South Africa is distinguished by a high amount of isomenthone, whilst the oil from Northwest Morocco has a large quantity of menthol.

This makes it evident to us how various settings and collecting periods affect the makeup of essential oils extracted from plants. Tables 2 and 3 provide a summary of these findings.

**Table 2:** Effects of geographical origin on *Pelargonium graveolens* (Thunb.) L' Hér aerial parts essential oil composition (Citronellol & Geraniol).

Country	First major compound Citronellol	Second major compound Geraniol	References
Egypt	29.90%	18.03%	^33^
India	33.6%	26.8%	^34^
Morocco	25.24%	23.36%	^35^
Serbia	24.54%	15.33%	^21^
Tajikistan	37.5%	6.0%	^22^
Tunisia	19.24%	15.30%	^17^

**Table 3:** Effects of geographical origin on *Pelargonium graveolens* (Thunb.) L'Hér aerial parts essential oil composition (Other compounds).

Country	First major compound	Second major compound	References
Algeria	Citronellol (30.2%)	Citronellyl formate (9.3%)	^36^
Bosnia and Herzegovina	Geraniol (27.5%)	Citronellol (19.0%)	^37^
Brazil	Geraniol (42.3%)	Linalool (16.4%)	^38^
Egypt	Citronellol (27.67%)	Cis-Menthone (10.23%)	^39^
Iran	*β*-Citronellol (36.4%)	Citronellyl formate (12.1%)	• ^40^
Iraq	Citronellyl isovalerate (10.41)	Menthol (9.61%)	^41^
Northwest Morocco	Menthol (14.06%-20.57%)	Isogeraniol (9.14%-15.47%)	^42^
Palestine	Citronellol (24.44%)	Citronellyl formate (15.63%)	^43^
South Africa	Isomenthone (84.0%)	Menthone (2.8%)	^20^

### Network pharmacology study

**Fig. 2:**
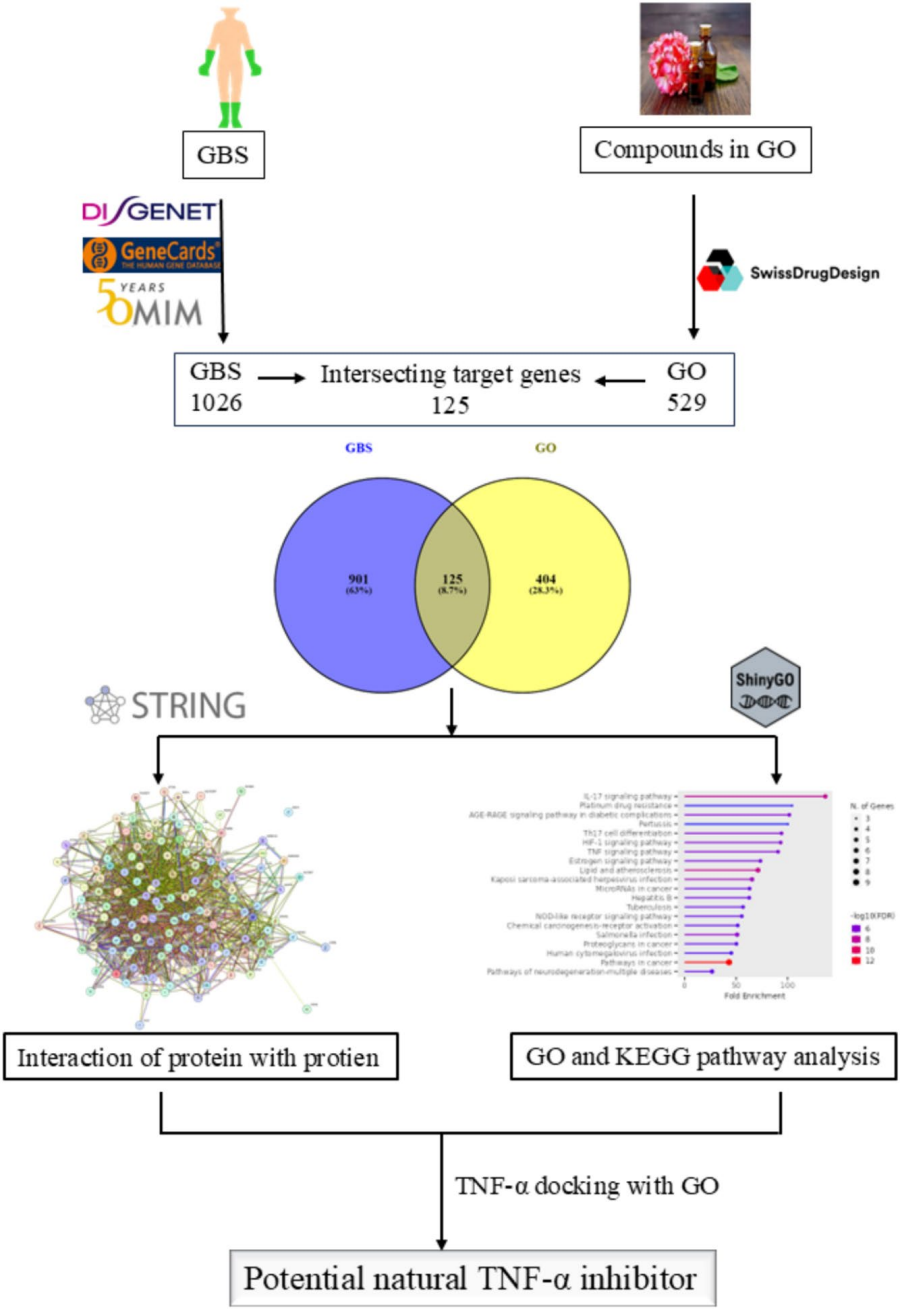
The workflow of TNF-α inhibitor prediction in GBS.

This study aimed to investigate the molecular mechanism of geranium oil in treating immune-inflammatory diseases of Guillain-Barré syndrome (GBS) and tumor necrosis factor-alpha (TNF-α) inhibitors (TNF) by using network pharmacology and molecular docking **(**Fig. 2**)**.

### Geranium oil active ingredient database establishment

All geranium oil ingredients were selected for database establishment **(**Table 1**)**.

### Potential target genes and the PPI network map geranium oil therapy for Guillain-Barré syndrome

**Fig. 3:**
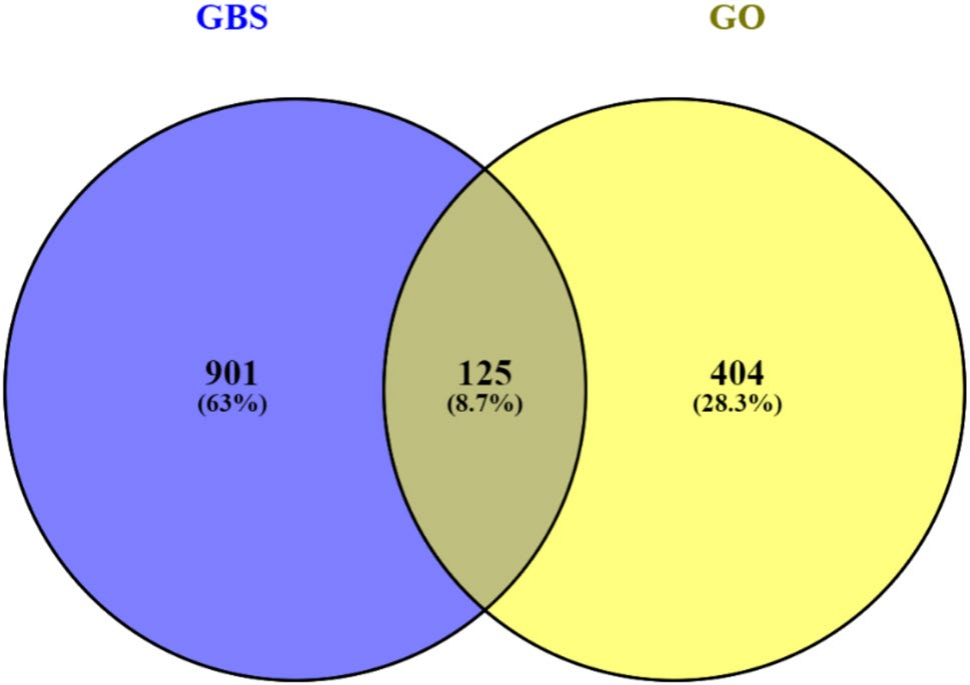
Potential target genes and PPI network map of geranium oil therapy for GBS.

The DiseGeNET, GeneCards, and OMIM databases were searched, yielding a total of 1026 Guillain-Barré syndrome (GBS)-related genes, after excluding duplicates. Similarly, 529 target genes were predicted for the active compounds of geranium oil using the SwissTargetPrediction database. To enhance confidence in the selected targets and reduce false positives, only targets with a high probability score (> 0.7 in SwissTargetPrediction) and a relevance score > 10 in GeneCards were retained. These two gene sets were intersected using the Venny 2.1 tool (http://bioinfogp.cnb.csic.es/tools/venny/) to identify 125 common potential therapeutic targets (Fig. 3 and Table 4).

**Table 4:** Potential target genes of GBS therapy for geranium oil.

No	Target	Symbol	No	Target	Symbol
1	Tumor necrosis factor	TNF	11	Telomerase reverse transcriptase	TERT
2	Beta-secretase 1	BACE1	12	Sex hormone binding globulin	SHBG
3	Acetylcholinesterase (Yt blood group)	ACHE	13	Amyloid beta precursor protein	APP
4	Angiotensin I converting enzyme	ACE	14	Monoamine oxidase B	MAOB
5	Tyrosinase	TYR	15	Glycogen synthase kinase 3 beta	GSK3B
6	Butyrylcholinesterase	BCHE	16	Coagulation factor II, thrombin	F2
7	Interleukin 6	IL6	17	KIT proto-oncogene, receptor tyrosine kinase	KIT
8	B-Raf proto-oncogene, serine/threonine kinase	BRAF	18	Peroxisome proliferator activated receptor gamma	PPARG
9	Synuclein alpha	SNCA	19	Matrix metallopeptidase 9	MMP9
10	Leucine rich repeat kinase 2	LRRK2	20	Solute carrier family 6 member 3	SLC6A3

**Fig. 4:**
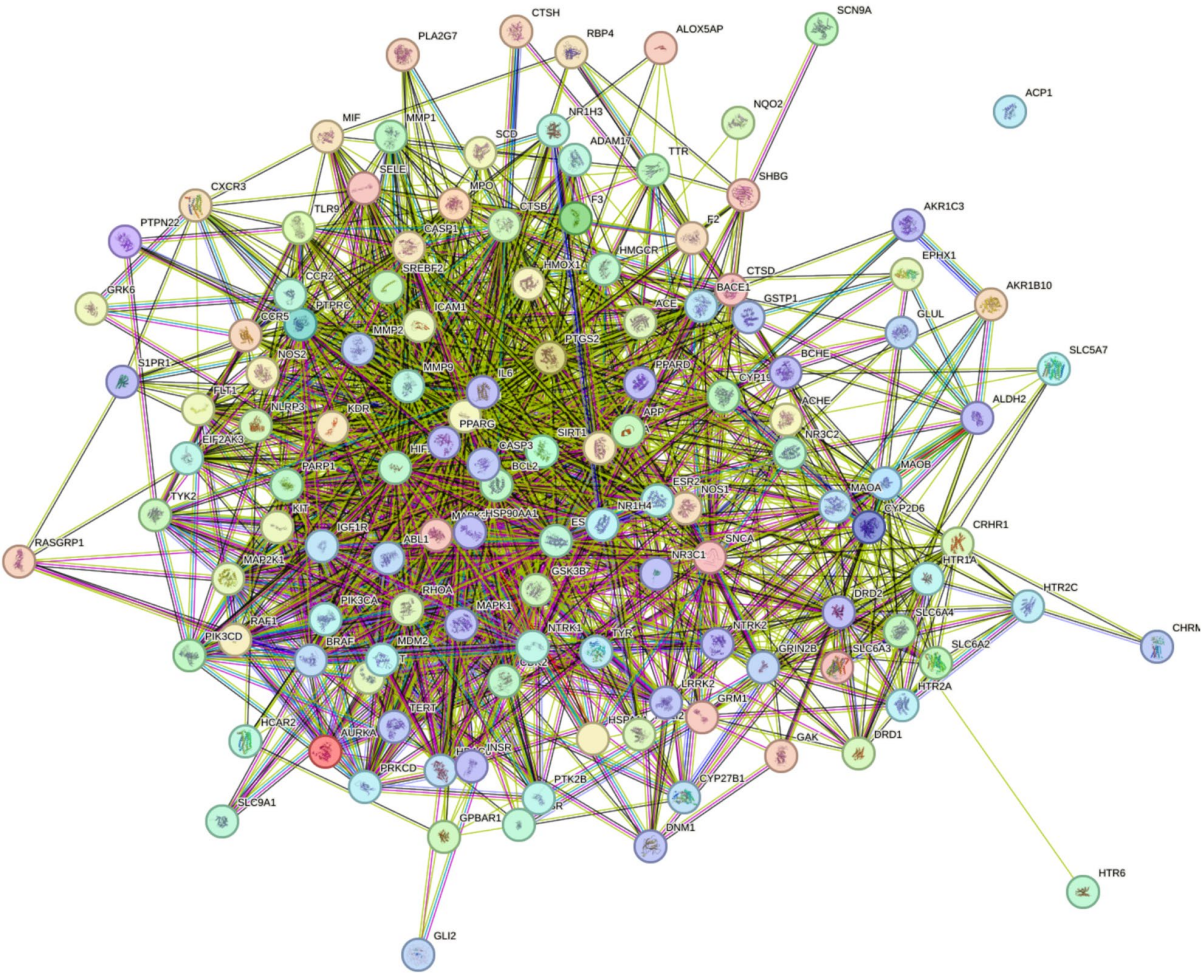
The Venny results of potential target genes of geranium oil therapy for GBS.

To explore the protein–protein interaction (PPI) relationships among these targets, we used the STRING database (https://string-db.org/) with the top ten intersected genes prioritized based on their connectivity scores in the network. The resulting PPI network was visualized and analyzed **(**Fig. 4**)**. The most interconnected or “hub” genes—likely playing key regulatory roles in GBS pathogenesis and geranium oil’s therapeutic effect—were identified as **TNF, BACE1, ACHE, ACE, TYR, BCHE, IL6, BRAF, and SNCA**. These genes have been reported in the literature to play roles in inflammatory responses, neurodegeneration, and neurotransmitter metabolism, which are all relevant to the immunopathology of GBS^44^.

#### GO and KEGG pathway enrichment analysis

**Fig. 5:**
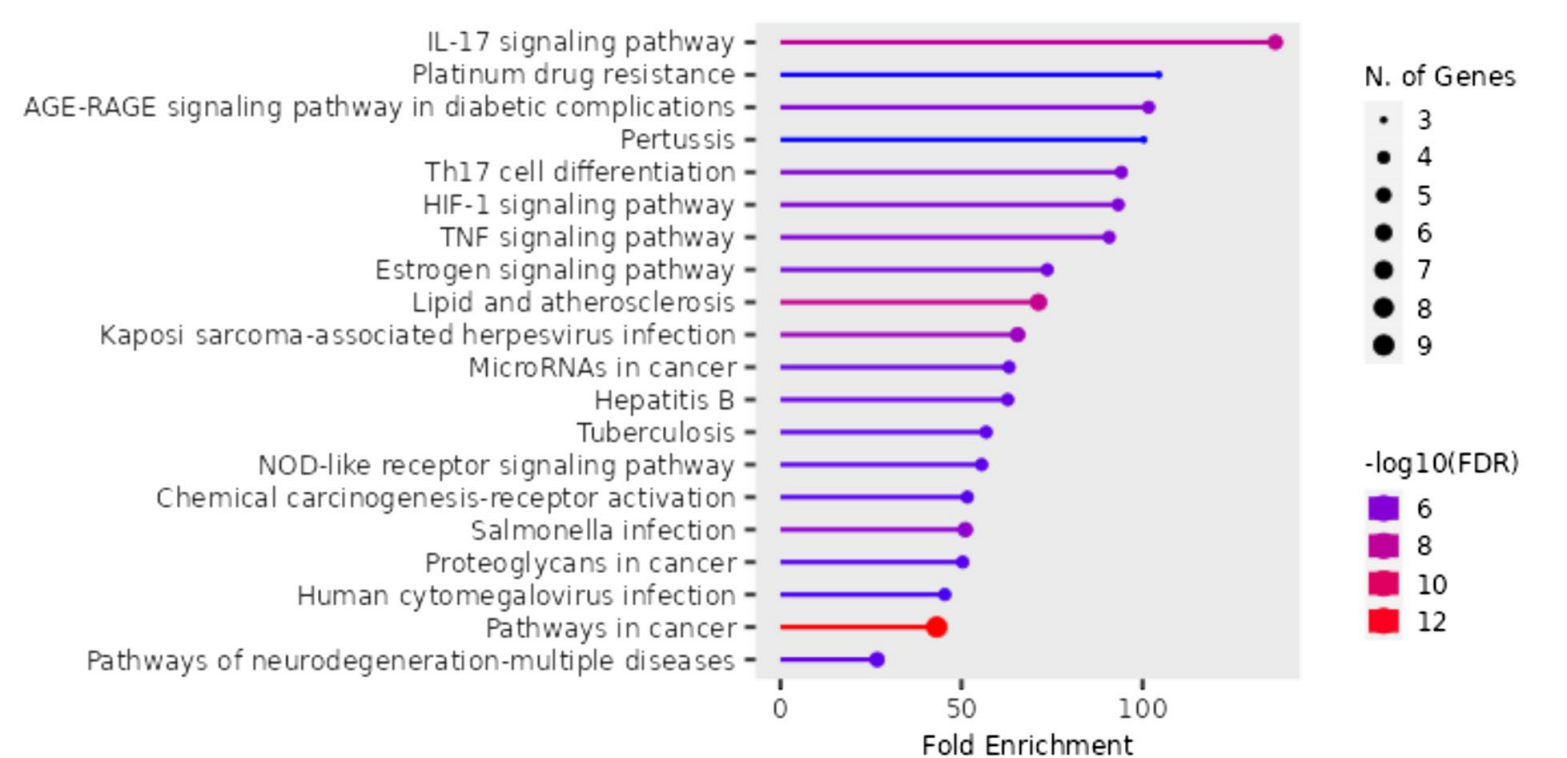
The top remarkably enriched geranium oil analyses for biological function of potential target genes of geranium oil in GBS.

**Fig. 6:**
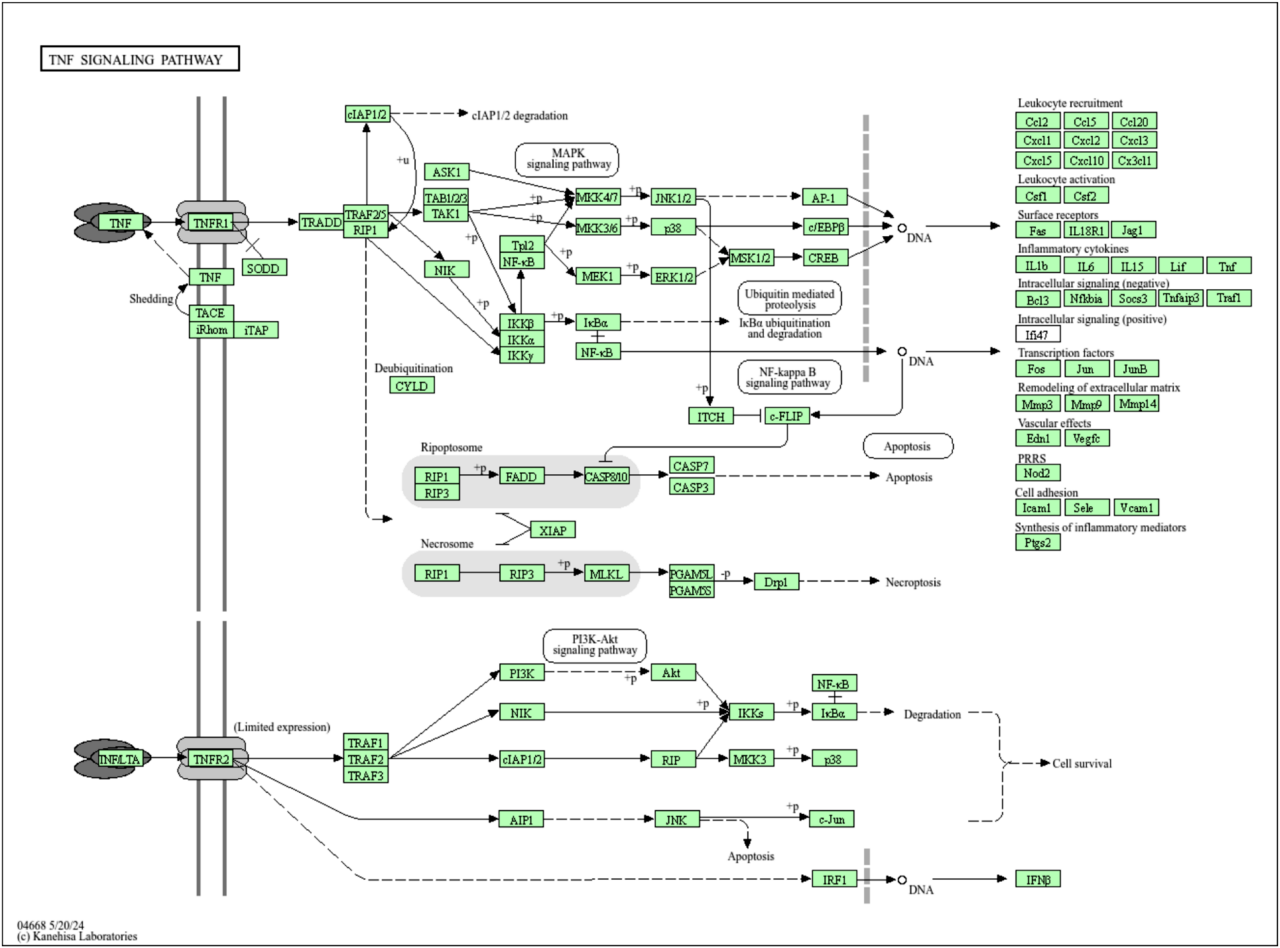
The TNF-α signaling pathway of potential target genes of geranium oil in GBS. Arrows indicate upstream and downstream relationships between genes.

The top markedly enriched gene biological function catalogs were selected for the generation of plots (Fig. 5). The biological functions of these genes mainly include the IL-17 signaling pathway, the TNF-α signaling pathway, and pathways of neurodegeneration-multiple diseases. These results suggest that geranium oil may influence the pathophysiology of GBS through the modulation of key immune-inflammatory pathways. Notably, IL-17 and TNF-α signaling pathways were significantly enriched. The active monoterpenes in geranium oil, such as geraniol and citronellol, have been reported to exhibit anti-inflammatory effects by downregulating pro-inflammatory cytokines including TNF-α and IL-6. It is therefore plausible that these components exert therapeutic effects in GBS by attenuating TNF-α-mediated complement activation and vesicular degeneration, as well as modulating IL-17-driven cytokine cascades. Further in vitro or in vivo studies are warranted to validate these mechanistic insights^45^. As shown in Fig. 6, many signaling pathways are closely associated with GBS, such as the TNF-α signaling pathway which induces the proliferation of B cells that activate complement, which leads to the formation of the MAC on the outer surface of Schwann cells, leading to initiation of vesicular degeneration, and invasion of myelin by macrophages.

### Binding capacity between the active ingredients and TNF-α by molecular docking study

Molecular docking was conducted to evaluate the binding affinity and interaction modes of oil components with TNF-α. To validate the docking protocol, a redocking experiment was performed using a known TNF-α inhibitor (e.g., SPD304). The root mean square deviation (RMSD) between the redocked and co-crystallized poses was calculated to confirm docking reliability. A control ligand was also included to benchmark binding affinity.

Docking was performed in a site-specific manner, focusing on the known active site of TNF-α, defined using the co-crystallized ligand from PDB entry 2az5. All compounds were docked into this defined pocket.

The results showed that citronellyl tiglate and geranyl tiglate had the highest binding affinity (ΔG = − 5.01 and − 5.14 kcal/mol, respectively), outperforming the control ligand and other oil components. These compounds formed key hydrogen bonds with residues such as Ser60, Gly121, and Tyr151, and engaged in hydrophobic interactions with Tyr59 and Tyr119.

Detailed 2D interaction maps revealed various non-covalent interactions, including hydrogen bonding, van der waals forces, and hydrophobic contacts, which are essential for the stabilization of the ligand-receptor complex. For example, geranyl tiglate exhibited π–π stacking with Tyr119 and hydrogen bonding with Ser60, suggesting a high potential for bioactivity.

**Fig. 7:**
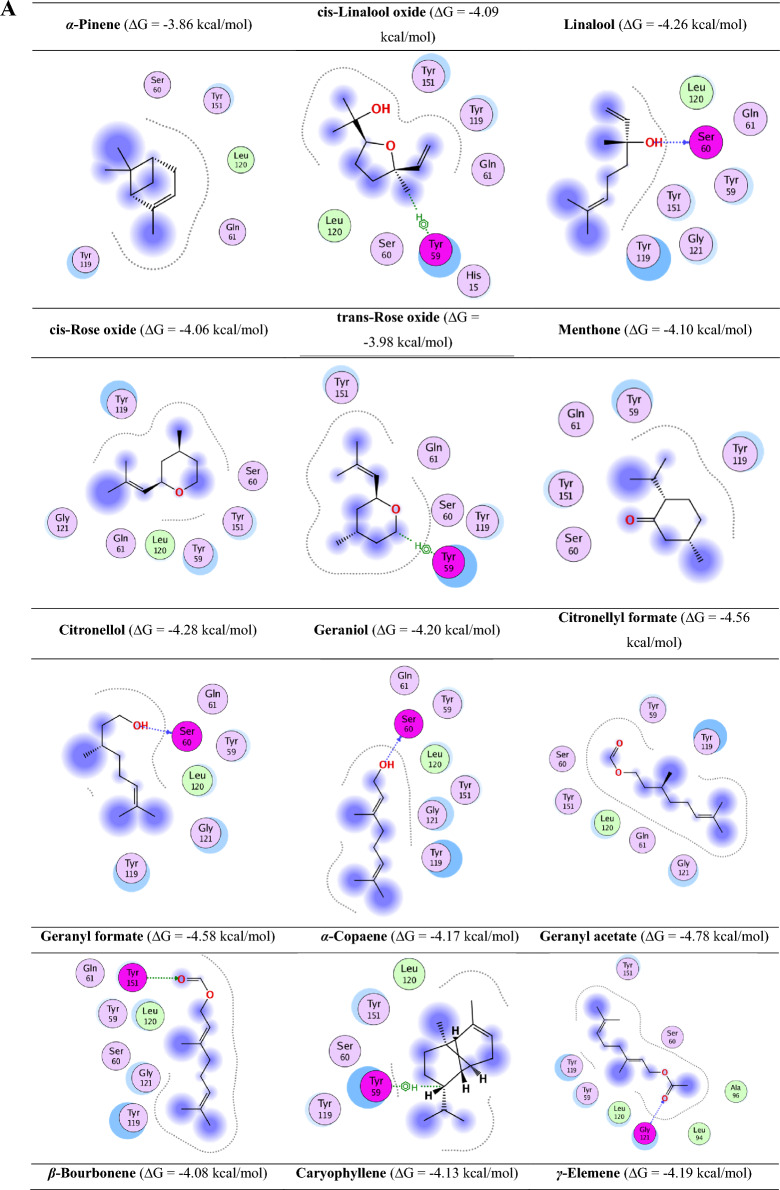

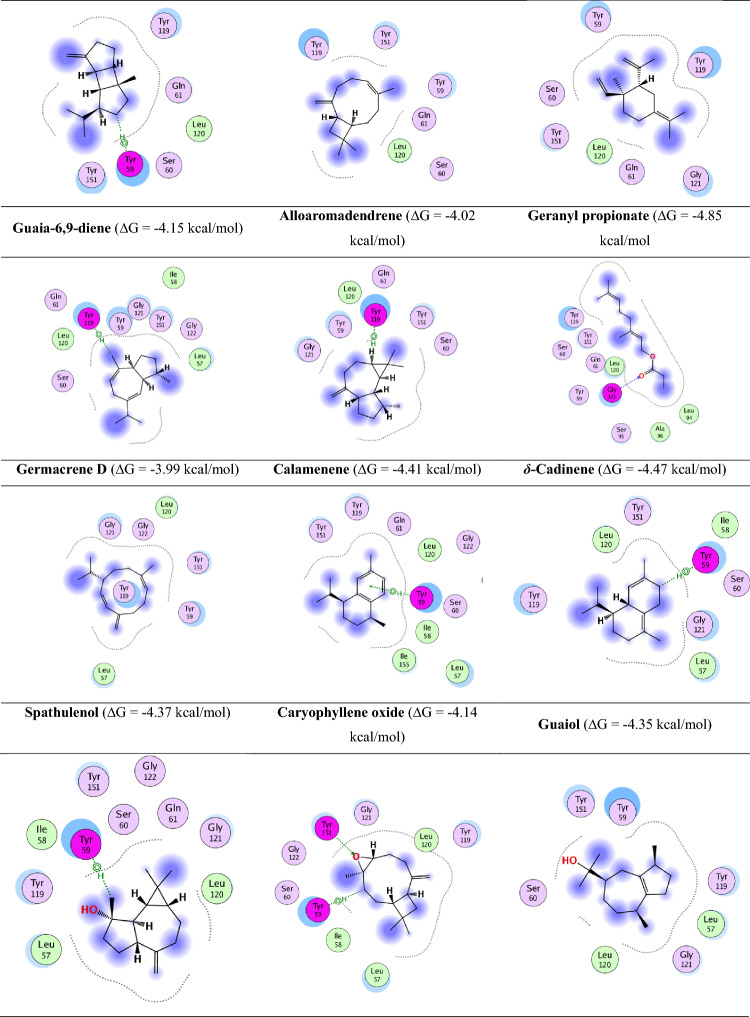

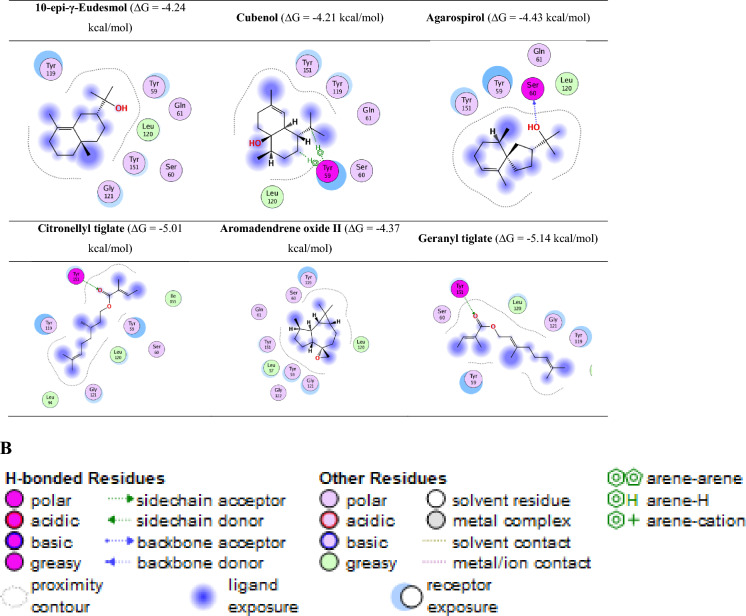
(**A**) The 2D map of the binding of all active ingredients and TNF-α active site and their binding scores. (**B**) Legend of the 2D mapping of the active ingredients’ interactions with TNF-α active site.

These results support the hypothesis that geranyl and citronellyl derivatives serve as promising scaffolds for natural TNF-α inhibitors. The 2D map of the binding of all active ingredients is shown in Fig. 7A and B.

### Evaluation of physicochemical, and pharmacokinetic prediction on active compounds

Physicochemical parameters (oral bioavailability) for both Lipinski’s and Veber rules were determined for oil components using Swiss ADME: a free web tool. The role of 5 states that a candidate meets the following requirements to be considered drug-like and orally active: molecular weight < 500 Da, octanol–water partition coefficient (log P), hydrogen-bond donor (HBD), and hydrogen bond acceptor (HBA). All of the identified phytochemical compounds displayed 0–1 violations of Lipinski’s rule, The logP values of compounds were less than 5, in the range of 2.2–4.5 and all of the oil compounds succeeded to pass through Veber rule by Topological polar surface area (TPSA) lower than 140 Å2. All of the compounds have high GIT absorption except *α*-pinene, caryophyllene, *γ*-elemene, guaia-6,9-diene and alloaromadendrene. The compounds also had BBB permeability except caryophyllene, *γ*-elemene and guaia-6,9-diene (Table 5).

**Table 5:** ADME properties for oil active ingredients.

Identified compounds	TPSA	Veber #violations	Consensus Log P	ESOL Log S	GI absorption	BBB permeant	Lipinski #violations	Bioavailability Score	PAINS #alerts	Leadlikeness #violations
*α*-Pinene	0	0	3.44	− 3.51	Low	Yes	1	0.55	0	2
cis-Linalool oxide	29.46	0	2.09	− 2.29	High	Yes	0	0.55	0	1
Linalool	20.23	0	2.66	− 2.4	High	Yes	0	0.55	0	1
cis-Rose oxide	9.23	0	2.66	− 2.55	High	Yes	0	0.55	0	1
trans-Rose oxide	9.23	0	2.65	− 2.55	High	Yes	0	0.55	0	1
Menthone	17.07	0	2.6	− 2.65	High	Yes	0	0.55	0	1
Citronellol	20.23	0	2.92	− 2.94	High	Yes	0	0.55	0	2
Geraniol	20.23	0	2.74	− 2.78	High	Yes	0	0.55	0	2
Citronellyl formate	26.3	0	3.18	− 3.36	High	Yes	0	0.55	0	2
Geranyl formate	26.3	0	3.06	− 3.19	High	Yes	0	0.55	0	2
*α*-Copaene	0	0	4.3	− 3.86	Low	Yes	1	0.55	0	2
Geranyl acetate	26.3	0	3.3	− 3.21	High	Yes	0	0.55	0	2
*β*-Bourbonene	0	0	4.4	− 4.01	Low	Yes	1	0.55	0	2
Caryophyllene	0	0	4.24	− 3.87	Low	No	1	0.55	0	2
*γ*-Elemene	0	0	4.56	− 4.35	Low	No	1	0.55	0	2
Guaia-6,9-diene	0	0	4.13	− 3.74	Low	No	1	0.55	0	2
Alloaromadendrene	0	0	4.34	− 4.07	Low	Yes	1	0.55	0	2
Geranyl propionate	26.3	0	3.67	− 3.52	High	Yes	0	0.55	0	2
Germacrene D	0	0	4.26	− 4.03	Low	No	1	0.55	0	2
Calamenene	0	0	4.57	− 4.55	Low	No	1	0.55	0	2
*δ*-Cadinene	0	0	4.14	− 3.43	Low	No	1	0.55	0	2
Spathulenol	20.23	0	3.3	− 3.17	High	Yes	0	0.55	0	1
Caryophyllene oxide	12.53	0	3.68	− 3.45	High	Yes	0	0.55	0	2
Guaiol	20.23	0	3.42	− 3.09	High	Yes	0	0.55	0	1
10-epi-*γ*-Eudesmol	20.23	0	3.57	− 3.29	High	Yes	0	0.55	0	1
Cubenol	20.23	0	3.52	− 3.48	High	Yes	0	0.55	0	2
Agarospirol	20.23	0	3.53	− 3.45	High	Yes	0	0.55	0	2
Citronellyl tiglate	26.3	0	4.21	− 3.92	High	Yes	0	0.55	0	3
Aromadendrene oxide II	12.53	0	3.54	− 3.52	High	Yes	0	0.55	0	2
Geranyl tiglate	26.3	0	3.54	− 3.52	High	Yes	0	0.55	0	2

Although the majority of the identified oil compounds demonstrated favorable drug-likeness based on Lipinski’s and Veber’s criteria and showed predicted BBB permeability, it is important to note that monoterpenes, due to their highly volatile and lipophilic nature, are often prone to rapid metabolic degradation in vivo. This metabolic instability can significantly reduce their half-life and systemic bioavailability, potentially limiting their therapeutic efficacy, particularly in targeting the central nervous system. Predicted BBB permeability should thus be interpreted with caution, as passive diffusion across the barrier does not necessarily guarantee central pharmacological activity in the absence of metabolic resilience. Further in vivo studies are essential to evaluate the pharmacokinetic behavior and CNS efficacy of these compounds under physiological conditions^46–48^.

The free web-based program, Osiris, has been used for toxicity prediction. Toxicity risks (mutagenicity, tumorigenicity, irritation, reproduction) were calculated by the methodology developed by Osiris (Table 6). The toxicity prediction using Osiris Property Explorer was shown in color codes. Green color shows the low toxicity tendency, yellow shows the moderate tendency, and red shows the high toxicity tendency. Most of the compounds revealed no mutagenicity, tumorigenicity or reproductive effects except for a few exceptions. However, some compounds showed a tendency to be irritants.

**Table 6:** Toxicity prediction for oil active ingredients.

Identified compounds	Mutagenicity	tumorgenicity	irritant	Reproductive effect
*α*-Pinene	Green	Green	Red	Green
cis-Linalool oxide	Green	Red	Orange	Green
Linalool	Red	Green	Red	Green
cis-Rose oxide	Green	Green	Red	Green
trans-Rose oxide	Green	Green	Red	Green
Menthone	Green	Green	Orange	Green
Citronellol	Green	Green	Red	Red
Geraniol	Green	Green	Red	Green
Citronellyl formate	Green	Green	Red	Green
Geranyl formate	Green	Green	Red	Green
*α*-Copaene	Green	Green	Red	Green
Geranyl acetate	Red	Red	Red	Orange
*β*-Bourbonene	Green	Green	Green	Green
Caryophyllene	Green	Green	Green	Green
*γ*-Elemene	Green	Green	Green	Green
Guaia-6,9-diene	Green	Green	Green	Green
Alloaromadendrene	Green	Red	Red	Green
Geranyl propionate	Green	Green	Red	Green
Germacrene D	Green	Green	Green	Green
Calamenene	Green	Green	Green	Green
*δ*-Cadinene	Green	Green	Green	Green
Spathulenol	Green	Red	Red	Green
Caryophyllene oxide	Green	Orange	Green	Orange
Guaiol	Green	Green	Green	Green
10-epi-*γ*-Eudesmol	Green	Green	Green	Green
Cubenol	Green	Green	Green	Green
Agarospirol	Green	Green	Green	Green
Citronellyl tiglate	Green	Green	Red	Green
Aromadendrene oxide II	Red	Red	Red	Green
Geranyl tiglate	Green	Green	Red	Green

## Conclusion

The oxygenated monoterpenes citronellol (26.74%) and geraniol (20.28%) are the two primary components of the essential oil of grown *Pelargonium graveolens* aerial parts, according to GC/MS analysis. The study claims that geranium oil aids in the treatment of GBS by encouraging many gene-based processes that aid in our understanding of the immune-modulatory systems that underlie GBS. The greatest and most stable binding affinities for TNF-α were found in geranyl and citronellyl derivatives, which may be the best material foundation for a natural TNF-α, according to the binding score. This encourages us to move forward with a future plan towards further *in-vivo* studies to validate these mechanistic ideas.

## Data Availability

The data that support the findings of this study are available from the corresponding author upon reasonable request.

## Abbreviations

GBS: Guillain-Barré syndrome
GO: Geranium oil
IL-6: Interleukin-6
PPI: Protein–Protein Interaction
TNF-α: Tumor necrosis factor alpha
